# Assessing Measurable Residual Disease in Chronic Myeloid Leukemia. BCR-ABL1 IS in the *Avant-Garde* of Molecular Hematology

**DOI:** 10.3389/fonc.2019.00863

**Published:** 2019-09-23

**Authors:** Vlad Moisoiu, Patric Teodorescu, Lorand Parajdi, Sergiu Pasca, Mihnea Zdrenghea, Delia Dima, Radu Precup, Ciprian Tomuleasa, Simona Soverini

**Affiliations:** ^1^Department of Hematology, Iuliu Hatieganu University of Medicine and Pharmacy, Cluj Napoca, Romania; ^2^Department of Hematology, Ion Chiricuta Clinical Research Center, Cluj Napoca, Romania; ^3^Department of Mathematics, Babes Bolyai University, Cluj Napoca, Romania; ^4^Department of Hematology, Research Center for Functional Genomics and Translational Medicine, Iuliu Hatieganu University of Medicine and Pharmacy, Cluj Napoca, Romania; ^5^Department of Experimental, Diagnostic and Specialty Medicine, Institute of Hematology L. and A. Seràgnoli, S. Orsola-Malpighi University Hospital, University of Bologna, Bologna, Italy

**Keywords:** chronic myeloid leukemia, mathematical modeling, BCR-ABL, IS, treatment free remission

## Abstract

Chronic myelogenous leukemia (CML) is a malignancy of the myeloid cell lineage characterized by a recurrent chromosomal abnormality: the Philadelphia chromosome, which results from the reciprocal translocation of the chromosomes 9 and 22. The Philadelphia chromosome contains a fusion gene called BCR-ABL1. The BCR-ABL1 codes for an aberrantly functioning tyrosine kinase that drives the malignant proliferation of the founding clone. The advent of tyrosine kinase inhibitors (TKI) represents a landmark in the treatment of CML, that has led to tremendous improvement in the remission and survival rates. Since the introduction of imatinib, the first TKI, several other TKI have been approved that further broadened the arsenal against CML. Patients treated with TKIs require sensitive monitoring of BCR-ABL1 transcripts with quantitative real-time polymerase chain reaction (qRT-PCT), which has become an essential part of managing patients with CML. In this review, we discuss the importance of the BCR-ABL1 assay, and we highlight the growing importance of BCR-ABL1 dynamics. We also introduce a mathematical correction for the BCR-ABL1 assay that could help homogenizing the use of the ABL1 as a control gene. Finally, we discuss the growing body of evidence concerning treatment-free remission. Along with the continuous improvement in the therapeutic arsenal against CML, the molecular monitoring of CML represents the *avant-garde* in the struggle to make CML a curable disease.

## Introduction

CML is a malignant proliferation driven by a characteristic fusion gene called BCR-ABL1 (1). The BCR-ABL1 gene results from the reciprocal translocation between chromosome 22 and 11 (the Philadelphia chromosome – Ph+) (2, 3). BCR-ABL1 codes a constitutionally active tyrosine kinase that inflicts growth advantage to the leukemic clone harboring the mutation. Based on cytology and cytogenetic studies, CML is further subdivided into chronic phase (CP), accelerated phase (AP), and blast phase (BP) CML (4). In CML, the presence of the Ph+ cells can be detected both in the peripheral blood and in the bone marrow using cytogenetic-based protocols that have been extensively used in the past for monitoring treatment response (5, 6). Moreover, cytogenetics is also important for detecting additional chromosomal alterations (ACA) such as trisomy eight, isochromosome 17q, second Ph, and trisomy 19 that bear prognostic value (7, 8).

The introduction of imatinib, the first tyrosine kinase inhibitor (TKI), has improved the treatment outcomes to such an extent that in many patients the disease burden decreases quickly below the detection limit of classical assays such as bone marrow cytogenetics (9–11). Therefore, the presence of measurable residual disease (MRD) must be monitored using quantitative polymerase chain reaction (qRT-PCR) of the BCR-ABL1 transcript, an assay which can detect as few as one malignant cell in 100.000 non-malignant ones (12–16). As the number of patients on long term therapy has increased, the MRD status evaluation has become increasingly important. Moreover, MRD can also be used as a convenient surrogate outcome in clinical trials (17).

The main goal of TKI therapy is to achieve a complete cytogenetic response (CCyR), defined as the lack of any detectable Ph+ cells in the bone marrow, within 12 months after starting treatment. CCyR is roughly equivalent to BCR-ABL1 IS ≤ 1%. The therapy for CP-CML is mainly represented by TKIs, which have improved the treatment outcomes compared to previous therapies. For instance, in the IRIS trial, a phase III trial that included more than 1,000 CML patients, and which eventually led to the approval of imatinib, the rate of complete cytogenetic response (CCyR) at 18 months in patients treated with imatinib was 76 vs. 15% for patients treated with interferon (IFN) plus cytarabine (10). Ever since, several other TKIs have been approved, namely dasatinib, nilotinib, bosutinib, and ponatinib (18–21). The front line TKI therapy for CP-CML includes imatinib and next-generation front-line TKIs: dasatinib, nilotinib and bosutinib. Next generation TKIs result in faster and deeper responses as well as in a lower risk of progression to advanced CML (13–15, 22). Nonetheless, randomized control trials showed no overall survival benefit for next-generation TKIs (13, 22). Next-generation TKIs are more appropriate for high-risk patients as well as in young and/or female patients for which treatment discontinuation is particularly important for fertility purposes. The TKI of choice for primary treatment is also dictated by the comorbidity profile of the patients. Hematopoietic stem cell transplantation (HSCT) is no longer recommended for CP-CML (23–25). Resistance to TKIs is determined by aberrant expressions of drug transporters (26, 27), plasma protein binding of TKIs (28–31), and mutations in the BCR-ABL1 tyrosine kinase domain that prevent the proper binding of the TKIs to their epitopes (31–36). There are more that 100 point mutations described so far, which have to be specifically treated with certain TKI: Y253H, E255K/V, or F359V/C/I mutations should be treated with dasatinib, F317L/V/I/C, T315A, or V299L mutations should be treated with nilotinib, while E255K/V, F317L/V/I/C, F359V/C/I, T315A, or Y253H mutations respond to ponatinib or omacetaxine (13, 37–44), the latter being a synthetic analog of a natural product isolated from the Japanese plum yew (45–49).

AP or BP CML should be treated by allogeneic HSCT after inducing remission with TKI and/or chemotherapy, regardless of whether the event occurred at diagnosis or progressed from CP-CML (50). Depending whether the BP is dominated by myeloid or lymphoid cells, acute myeloid leukemia or acute lymphocytic leukemia type protocols should be employed (51–53). Accelerated phase CML patients that progressed on TKI therapy also benefit from omacetaxine (54, 55).

## The BCR-ABL1 Is Assay

In the era of TKIs, for most patients only qRT-PCR is sensitive enough for detecting residual disease in the long term. CML burden monitoring via qRT-PCR was considered even before the advent of TKI (56, 57), but it was the unprecedented response to TKIs that pushed toward the implementation and standardization of the BCR-ABL1 assay.

The implementation of the BCR-ABL1 assay in the clinical practice was also aided by the development of an international scale (IS). The IS reference is represented by the median value of 30 samples collected from patients with newly diagnosed CP CML who were enrolled in the IRIS trial (58). Since these samples have been exhausted, the calibration to IS is currently achieved by either exchanging a set of samples with reference laboratories that maintain strict quality control or by using the World Health Organization (WHO) primary reference standard of four reference reagents (stably stored lyophilized cell line mixtures) assigned a fixed BCR-ABL1 IS value (10, 1, 0.1, 0.01%) BCR-ABL1 RNA (59, 60). It should be noted that BCR-ABL1 IS = 100% is just a reference ratio between BCR-ABL1 mRNA and control mRNA and it does not mean that the number of BCR-ABL1 mRNA is equal to that of control mRNAs. Alternatively, it is customary to express response as the log reduction compared to BCR-ABL1 IS = 100%. For example, a BCR-ABL1 IS = 0.1% represents a log three reduction compared to baseline (MR 3.0).

The reference gene for the BCR-ABL1 assay is usually represented by the ABL1. The ABL1 gene was chosen through an international initiative out of a panel of 14 candidate genes and has since become the standard control gene for most laboratories performing BCR-ABL1 quantification (61). The major criteria for selecting the control gene were the following: (i) absence of pseudogenes; (ii) not very high or very low expression; (iii) no significantly different expression levels between normal and leukemic samples; (iv) no significantly different expression levels between peripheral blood and bone marrow. Despite fulfilling these criteria, the ABL gene has an important drawback in that the amplicons also include the BCR-ABL1 gene. Thus, when ABL is used as a reference, the BCR-ABL1 assay expresses not the BCR-ABL1/ABL ratio but the proportion of BCR-ABL1 transcripts, i.e., BCR-ABL1/BCR-ABL1+ABL. The two are approximately equal however when BCR-ABL1 < < ABL. In other words, the BCR-ABL1/BCR-ABL1+ABL is an acceptable proxy for the BCR-ABL1/ABL for BCR-ABL1 IS values below 10%. We will show below that this bias can be mitigated based on a mathematical correction, allowing the ABL1 gene to be used as control even for BCR-ABL1 IS values >10%.

## The Role of BCR-ABL1 is in Monitoring CML

**Figure 1:**
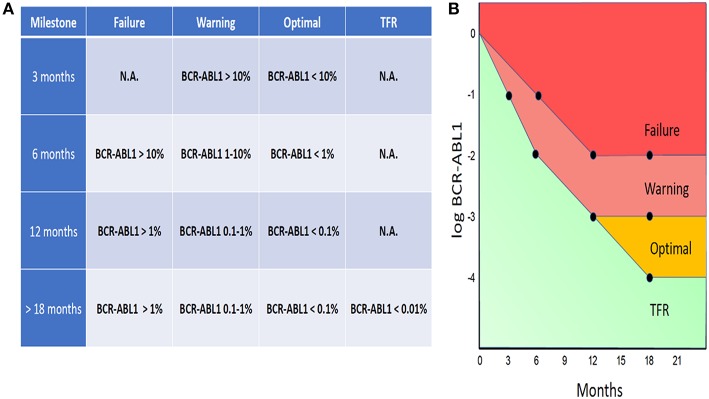
ELN-based guidelines threshold values, labeled as Optimal, Warning, or Failure **(A)**. BCR-ABL1 IS “trajectories” designated as Failure, Warning, or Optimal **(B)**.

The evidence for the prognostic and predictive value of BCR-ABL1 IS is compelling and serial assessment of BCR-ABL1 IS is now an integral part of CML guidelines. According to the ELN guidelines, BCR-ABL1 testing should be performed every 3 months for 2 years and then every 3–6 months thereafter, provided that the patient fulfilled all required milestones (4). In the case of each milestone, the ELN guidelines define threshold values, based on which the response to the treatment can be labeled as *Optimal, Warning*, or *Failure* (Figure 1A).

By uniting the threshold values defined by the ELN guidelines, there is a whole spectrum of BCR-ABL1 IS “trajectories” that can be designated as Failure, Warning, or Optimal (Figure 1B). The ELN guidelines also define special conditions for attempting treatment-free remission (TFR) (see below the discussion o TFR).

According to the ELN guidelines, the optimal response threshold at 12 months is BCR-ABL1 IS <0.1% [also called major molecular response (MMR)]. Achieving MMR is associated with a negligible risk of disease progression compared to patients that failed to achieve this milestone (62–66). However, the prognostic significance of MMR has been challenged by the lack of statistical significance when adjusting for multiple comparisons (62, 64, 65). Thus, there is an ongoing research regarding the appropriate management of patients having BCR-ABL1 IS between 1 and 0.1% in the long run. This issue has been recently tackled by the German CML Study IV group, which showed evidence suggesting that the optimal waiting time for patients to achieve MMR is about 2.5 years (67), after which a change in the treatment should be considered.

Beyond MMR, achieving deeper remission is an important concept as well. Deep molecular remission (DMR) is variably defined as either BCR-ABL1 IS <0.01% (MR 4.0) or BCR-ABL IS <0.0032% (MR 4.5). In the German CML study IV, the life expectancy of patients with MR4 or MR4.5 was the same as that of age-matched population (68). The same study also showed that among 792 patients who achieved MR4, only four patients (5%) displayed disease progression. MMR and DMR also corelate with improved EFS, PFS and OS (69, 70). Thus, DMR has been taken into consideration as a treatment goal in itself and a proper surrogate endpoint in clinical trials (17).

The absence of any detectable BCR-ABL1 transcripts is termed complete molecular remission (CMR) or molecular undetectable leukemia and it corelates with better outcomes in terms of event-free survival (EFS), progression-free survival (PFS), and overall survival (OS) (69, 70). Designating CMR must always be accompanied by the sensitivity and the control gene of the assay. However, one of the most important implication of DMR and CMR regards the achievement of treatment-free remission, which was shown to be feasible in selected patients.

At the other end of the spectrum, there is an ongoing research regarding the role of early molecular response (EMR) as a means of predicting outcomes and treatment responses. EMR is defined as BCR-ABL1 IS <10% at 3 months. Achieving EMR is associated with improved PFS and OS compared to patients not achieving this milestone (71–73).

## Dynamical Parameters

Several studies have shown that the dynamics of the BCR-ABL1 values can be used for devising independent predictors of outcome (74–76). For instance, Branford et al. have shown that for patients with >10% BCR-ABL1 IS after 3 months of imatinib, the rate of BCR-ABL1 decline at 3 months defines distinct prognostic subgroups. Patients with a BCR-ABL1 decline by at least one-half at 76 days (74 out of 95 patients, 78%) had significantly superior outcomes compared with the patients (21 out of 95 patients, 22%), where the halving time was >76 days in terms of OS, PFS EFS, and MMR (77).

Similarly, Hanfstein et al. showed that that the velocity of the BCR-ABL1 decay successfully predicts outcome and that a 0.35-fold reduction of BCR-ABL1 compared to baseline levels at 3 months (0.46-log reduction, that is, roughly half-log) defines distinct risk subgroups in terms of overall survival (78). In order to calculate the fold decrease compared to baseline, the authors had to use beta glucuronidase (GUSB) as control gene, since many BCR-ABL1 IS values were above 10% at the time of diagnosis. Thus, the authors suggested that implementing dynamical parameters will require abandoning ABL1 as the control gene and using alternative control genes such as GUSB. However, we believe that the substituting ABL1 as the control gene is not required and that the situation can be mitigated using a mathematical transformation.

## Mathematical Correction of BCR-ABL1 >10%

**Figure 2:**
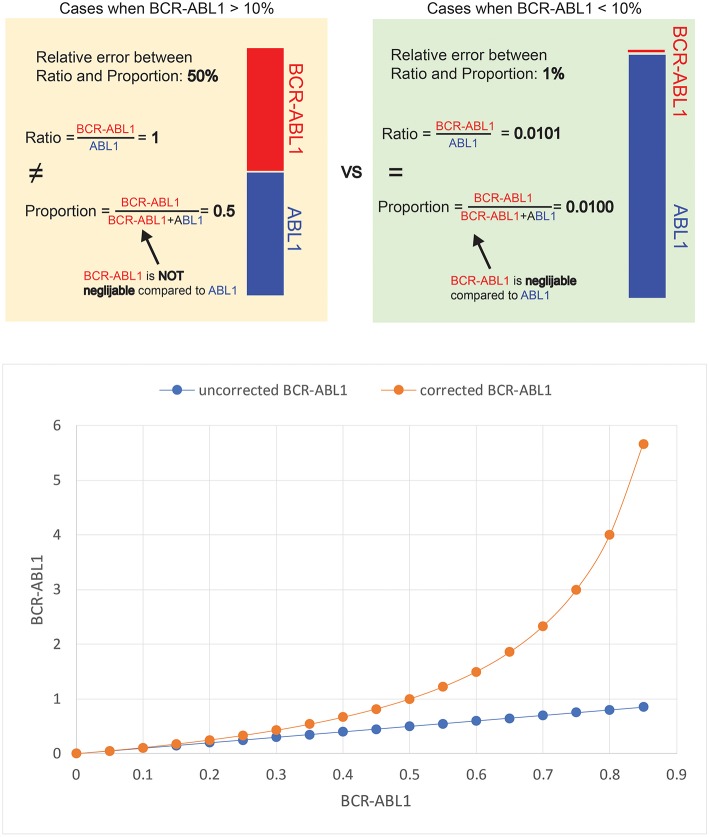
Scenario when out of the total number of BCRABL1 plus ABL1 transcripts, the BCR-ABL1 transcripts represent 1 part, and the ABL1 transcripts represent 99 parts.

The reasoning behind the mathematical transformation lies in the distinction between ratio and proportion. The aim of the BCR-ABL1 assay is to calculate the ratio between the BCR-ABL1 transcripts and the ABL1 transcripts (i.e., BCR-ABL1/ABL1). However, the actual results of the assay are represented by the proportion of BCR-ABL1 transcripts from the total number of BCR-ABL1 plus ABL1 transcripts (i.e., BCR-ABL1/BCR-ABL1+ABL1). The distinction between ratio and proportion is unimportant when the results of the assay is small, as it is most often the case in clinical practice due to the excellent response to TKIs. In a situation when out of the total number of BCR-ABL1 plus ABL1 transcripts, the BCR-ABL1 transcripts represent 1 part, and the ABL1 transcripts represent 99 parts (Figure 2 top, green box). In this case, the proportion of BCR-ABL1 transcripts is 1% (1/99+1), while the ratio between BCR-ABL1 transcripts and ABL1 transcripts is 1.01% (1/99). Thus, the proportion and ratio of BCR-ABL1 transcripts are almost equal, since the number of BCR-ABL1 transcripts is negligible compared to the ABL1 transcripts.

Still, a sample from a patient might have an equal number of BCR-ABL1 transcripts and ABL1 transcripts (Figure 2 top, yellow box). The BCR-ABL1 transcripts represent 50 parts and the ABL1 transcripts also represent 50 parts. In this case, the BCR-ABL1 represent 50% of the total number of BCR-ABL1 plus ABL1 transcripts (50/50+50), while the ratio between the two is 100% (50/50). Fifty percent differs significantly from 100%, whereas 1% is very close to 1.01%. The threshold for deciding whether the proportion of BCR-ABL1 transcripts is an acceptable proxy for the ration between BCR-ABL1 and ABL1 transcripts has been arbitrarily set to BCR-ABL1 IS = 10%. The relation between the mathematically corrected and experimental BCR-ABL1 values is shown in Figure 2 (below). The red curve in Figure 2 is the depiction of Equation (1) and it can be considered a nomogram for correcting the results of the BCR-ABL1 assay. The correction can be applied for the whole range of experimental BCR-ABL1 results, but the correction will be significant only when the experimental results of the BCR-ABL1 assay are large (i.e., BCR-ABL1 IS >10%).

The mathematical transformation proposed here is a method of extracting the ratio of two numbers when the proportion of one of the numbers is known. In other words, we describe a strategy of retrieving the BCR-ABL1/ABL1 from the BCR-ABL1/(ABL1 + BCR-ABL1) ratio using a mathematical correction, such that the ABL1 control gene could be used even for BCA-ABL1 IS values higher than 10% (see below).

It can be showed that the BCR-ABL1/ABL1 ratio (*X*) can be extracted from the BCR-ABL1/(ABL + BCR-ABL1) proportion (*Y*) by the following transformation (see Supplementary Materials for the deduction of the formula):

(1) $$\begin{array}{l} X=\frac{Y}{1-y} \end{array}$$

The relation between the mathematically corrected and experimental BCR-ABL1 values is shown in Figure 2 (bottom). The red curve in Figure 2 is the depiction of Equation (1) and it can be considered a nomogram for correcting the results of the BCR-ABL1 assay. The correction can be applied for the whole range of experimental BCR-ABL1 results, but the correction will be significant only when the experimental results of the BCR-ABL1 assay are large (i.e., BCR-ABL1 IS >10%).

Thus, we propose a mathematical correction to help removing the bias caused by counting both malignant and non-malignant transcripts in the denominator of the BCR-ABL1 assay when using ABL1 as a control gene. This corrected BCR-ABL1 values represent the hypothetical results of the BCR-ABL1 assay is the ABL1 amplicon would not be contained in the BCR-ABL1 amplicon. For further explanations on the bias caused by the ABL1 gene, see the argument described in the study by Hanfstein et al. for employing an alternative gene as control (namely GUSB) (78).

With increasing evidence in support of the predictive and prognostic value of dynamical parameters, it is expected that situations when calculation of BCR-ABL1 IS values higher 10% is required will be more and more often. Using the mathematical transformation presented here, BCR-ABL1 transcripts values above 10% could be reliably calculated without changing ABL1 as the control gene, thus spearing laboratories from the effort of redesigning their assays and helping to translate dynamical parameters in the clinical setting.

## Treatment-Free Remission

The possibility to achieve treatment free remission (TFR), a term defined as sustained MMR after TKI treatment discontinuation, was put forward more than a decade ago (79–82). Since then, the concept was validated in several trials involving patients who were on sustained DMR. The results showed that treatment discontinuation leads to sustained MMR in around 50% of patients (83). Consequently, in the United States the Food and Drug Administration (FDA) has updated the label of nilotinib to reflect the possibility to achieve TFR.

The ELN guidelines consider that discontinuing therapy is feasible in low-risk CP CML patients achieving MR4.5 that remained in DMR (at least MR4.0) for >2 years after having been on first line TKI therapy at least 5 years. Attempting treatment discontinuation also requires no history of accelerated or blast phase CML and no history of TKI treatment resistance, among others. Access to reliable and quick BCR-ABL1 IS assay with a sensitivity of at least 4.5 MRD is also important.

Interestingly, the mechanism behind TFR is not yet fully understood. Several studies brought strong evidence for the fact that TKI therapy does not directly eliminate CML stem cells (84, 85). Another possibility is that TKI therapy eliminates CML stem cells indirectly, or that the immune surveillance can take care of MRD. This later hypothesis is supported by several studies linking successful TKI discontinuation with mechanistic insights of the immune system such as an increased proportion of mature NK cells or decreased PD-1 and immune suppressors (86, 87). However, a direct cause-effect relation is yet to be proven. Moreover, epidemiologic studies in immunocompromised patients failed to attribute immunity a pivotal role in CML progression (88).

Another possible explanation for successful TFR is that the time for developing a full-blown CML can take several years and that the process could be delayed by stochastic events (89, 90). As data from TFR clinical trials mature, the lengths of remission after treatment discontinuation will certainly bring more insights regarding the role of disease latency. In the meanwhile, treatment-free remission continues to be looked upon with care outside the setting of clinical trials. The manuscript has its limitations and this correction typically is more impactful for transcript levels above 10% and the question is of the clinical applicability as below this is more meaningful disease response criteria of major molecular remission, deep molecular remission, and complete molecular remissions.

## Data Availability

The raw data supporting the conclusions of this manuscript will be made available by the authors, without undue reservation, to any qualified researcher.

## Author Contributions

All authors contributed in the design of the hypothesis. VM and CT wrote the manuscript. SS supervised the work. MZ significantly contributed to the revision of the initial submitted manuscript, figures as well as in re-designing and adding new, important paragraphs to the manuscript, and thus contributing to the acceptance of the manuscript.

### Conflict of Interest Statement

The authors declare that the research was conducted in the absence of any commercial or financial relationships that could be construed as a potential conflict of interest.
