# Supplementary material for: Assessing Measurable Residual Disease in Chronic Myeloid Leukemia. BCR-ABL1 IS in the Avant-Garde of Molecular Hematology
Source: Front Oncol. 2019 Sep 23;9:863. doi: 10.3389/fonc.2019.00863 (PMC6768007; doi:10.3389/fonc.2019.00863)
Supplement: Supplementary file 1 [file Data_Sheet_1.docx]

**Supplementary materials**

Let X denote the results of the BCR-ABL1 assay using ABL1 as the control gene (uncorrected values). In this case:

X=$\frac{BCR-ABL1}{ABL1+BCR-ABL1}$ (1)

On the other hand, let Y denote the hypothetical result of the BCR-ABL1 assay if the ABL1 amplicon would not suffer from the drawback of being common to both the ABL1 and the BCR-ABL1 gene (corrected value). Thus:

Y=$\frac{BCR-ABL1}{ABL1}$

The mathematical correction described in this study is a way of estimating Y from X. To this end, divide both the numerator and the denominator of the righthand side in Equation 1, yielding the following expression:

X=$\frac{\frac{BCR-ABL1}{ABL1}}{1+\frac{BCR-ABL1}{ABL1}}$ (2)

It can be seen that in this case, the desired ration between BCR-ABL1 and ABL1 (namely Y) becomes apparent. Thus, Equation 2 can be rewritten in the following form in terms of X (uncorrected) and Y (corrected) values:

X=$\frac{Y}{1+Y}$ (3)

By expressing Y from Equation 3, the following formula results:

X=$\frac{Y}{1-y}$ (4)

Thus, Equation 4 represents a mathematical correction which eliminates the undesired property of the ABL1 control gene of containing both malignant (BCR-ABL1) and non-malignant transcripts (ABL1).
